# New Process for Making Fluid Extracts

**Published:** 1865-10

**Authors:** 


### NEW PROCESS FOR MAKING FLUID EXTRACTS.

This invention relates to an improved process for producing that class of extracts which are made so that a certain amount of liquid shall represent, pound for pound, medically, the same quantity of crude drug, and which are generally obtained by extracting with a large excess of liquid and evaporating down to the desired strength. The menstrua used for making extracts are usually of an ethereal or volatile nature, such as alcohol of various strengths, and their strength changed by evaporation, as they are exposed for a longer or shorter period to the open atmosphere. If such menstruum is poured over a certain drug, it dissolves and extracts more or less of the soluble parts of the same, according to its strength; if the same liquid has to be poured repeatedly over the same drug, it loses its strength alcoholically, and some of those portions first dissolved are precipitated, and an imperfect extract is the result. The value of the extract being determined by its alcoholic strength when finished, the same or similar reasons render it objectionable to subject the extract when first obtained to the evaporating process, for by this process the volatile or spirituous part of the menstruum is first evaporated, and the weaker liquid is not capable of keeping in solution many of those parts of the drug which has originally been dissolved in the extract.

The objections are obviated by my process, which is carried out in the following manner:—I first weigh off a quantity of drug, and the same quantity or more by weight of the menstruum or liquid by means of which the extract has to be made, a little more of the menstruum being required as a little moisture is left in at the last pressing. The drug being ground to a proper fineness, is then damped with a small portion of the liquid and subjected to heavy pressure (say from 800 to 1,000 tons); already all the liquid, or nearly so, together with such parts of the drug which have dissolved in the same, is expressed. A fresh portion of the liquid is then sprinkled over the drug, a little time being allowed for the liquid to dissolve the soluble parts of the drug, and the same process of pressing repeated until the whole quantity of the liquid is used up, and the drug is completely exhausted and the required measure obtained. By this process an extract is obtained which represents, pound by pound, the crude drug. The drug is perfectly extracted, and the menstruum preserves its original strength throughout, so that the same is capable of retaining in solution all those parts which are dissolved during the various stages of the process. Furthermore, by my process the tedious and expensive process by evaporation is dispensed with, and concentrated fluid extracts of any de-description can be produced cheaper and better than by any process heretofore applied, and as the application of heat is entirely avoided, the preparation does not receive the injury by heat that all such preparations are liable to if heat is applied to them, no matter how carefully done or moderate the degree of temperature, and furthermore, the changes thereby of strength of solvent are avoided. [From United States Patent, taken out by Mr. Spencer Thomas.]

